# Genomic analysis in chemotherapy-naïve prostate cancer prior to PSMA-targeted treatment

**DOI:** 10.3389/fonc.2026.1741080

**Published:** 2026-02-04

**Authors:** Mariam Amghar, Mareike Roscher, Tobias Rausch, Hilal Özgür, Ulrike Bauder-Wüst, Frank Bruchertseifer, Alfred Morgenstern, Vladimír Beneš, Clemens Kratochwil, Martina Benešová-Schäfer

**Affiliations:** 1 Junior Research Group Translational Radiotheranostics, German Cancer Research Center (DKFZ), Heidelberg, Germany; 2 Service Unit Radiopharmaceuticals und Preclinical Studies, German Cancer Research Center (DKFZ), Heidelberg, Germany; 3 Genomics Core Facility, European Molecular Biology Laboratory (EMBL), Heidelberg, Germany; 4 European Commission, Joint Research Centre (JRC), Karlsruhe, Germany; 5 Department of Nuclear Medicine, University Hospital, Heidelberg, Germany

**Keywords:** cfDNA, chemotherapy-naïve, copy number alternations, mCRPC, PSMA, radiopharmaceutical therapy, tandem actinium-lutetium therapy

## Abstract

**Introduction:**

Chemotherapy is typically administered prior to consideration of tandem [^225^Ac]Ac-/[^177^Lu]Lu-PSMA-617 therapy in metastatic castration-resistant prostate cancer (mCRPC), making chemotherapy-naïve patients who undergo tandem radionuclide treatment extremely rare. The genomic mechanisms dictating response and resistance to prostate-specific membrane antigen–radiopharmaceutical therapy (PSMA-RPT) in this setting remain unclear. While tandem therapy is expanding for aggressive disease, baseline genomic predictors of treatment outcomes are not well defined. We present rare chemotherapy-naïve mCRPC cases treated with tandem PSMA-RPT and explore their molecular characteristics through plasma circulating tumor DNA (ctDNA).

**Methods:**

Blood samples were obtained from mCRPC patients receiving [^225^Ac]Ac-/[^177^Lu]Lu-PSMA-617 therapy. Cell-free DNA (cfDNA) was isolated and analyzed using ultra-low pass whole-genome sequencing (ULP-WGS). Genome-wide copy number alterations (CNAs) and tumor fraction (TFx) were inferred with the ichorCNA algorithm.

**Results:**

This case series included five chemotherapy-naïve patients—four with baseline characterization and one with longitudinal follow-up—providing a rare window into cfDNA CNAs at treatment initiation. Recurrent alterations included amplifications in chromosomes 1q, 7q, and 8q, and losses in 8p. Additional events such as 12q amplification and partial 9q gain were also observed. In Patient 5, serial cfDNA analysis demonstrated stable 8p loss and 8q gain across multiple treatment cycles, despite clinical progression, suggesting clonally persistent genomic drivers.

**Discussion:**

Baseline cfDNA CNA profiling in chemotherapy-naïve mCRPC reveals recurrent chromosomal imbalances—particularly 8p loss and 8q gain—that may represent intrinsic, stable features of advanced disease. These findings highlight the exploratory potential of cfDNA-based genomics in rare PSMA-RPT cohorts.

## Graphical Abstract

**Figure:**
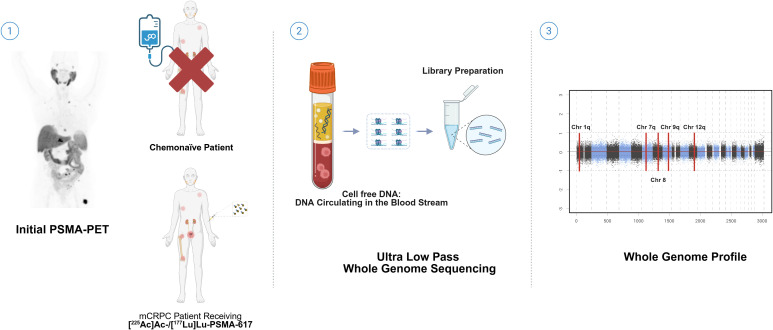

## 1 Introduction

Following the demonstration of prolonged overall survival (OS) in the VISION trial (1–3), the Food and Drug Administration and the European Medicines Agency approved [^177^Lu]Lu-PSMA-617 (Pluvicto^®^) in 2022 for treating metastatic castration-resistant prostate cancer (mCRPC) patients previously treated with at least one line of androgen receptor pathway inhibitors and taxane chemotherapy. This approval marked a significant advancement in the mCRPC treatment landscape. More recently, in March 2025, the Food and Drug Administration approved an expanded indication for Pluvicto^®^, allowing its use already in PSMA-positive mCRPC patients who have progressed following treatment with androgen receptor pathway inhibitors and are candidates for delaying chemotherapy (4–7). As prostate-specific membrane antigen-radiopharmaceutical therapy (PSMA-RPT) gains ground, experimental tandem treatment—combining lutetium- and actinium-labelled PSMA ligands—is increasingly considered for patients with aggressive disease biology or suboptimal response to lutetium alone (8–10). However, the genomic determinants of response and resistance to radioligand therapy remain poorly understood, particularly in chemotherapy-naïve patients. This population is rare and underrepresented, as chemotherapy is commonly incorporated into the clinical management of mCRPC before experimental approaches are warranted. This study sheds a light in the characterization of chemotherapy-naïve patients upon receiving [^225^Ac]Ac-/[^177^Lu]Lu-PSMA-617 therapy. Identifying genomic resistance signatures in heavily pre-treated patients specific to RPT is inherently challenging: prior cytotoxic therapies damage DNA, induce mutational scarring, and obscure disease-intrinsic genomic alterations (11, 12). Circulating tumor DNA (ctDNA) analysis offers a non-invasive window into tumor biology, capturing copy number alterations, tumor burden, and real-time treatment dynamics —while eliminating the need for biopsies from multiple metastatic sites (13–17). This exploratory study investigates the baseline molecular profiles of five chemotherapy-naïve mCRPC patients treated with [^225^Ac]Ac-/[^177^Lu]Lu-PSMA-617 therapy to describe the molecular landscape the genomic alterations observed. Using ultra-low-pass whole-genome sequencing (ULP-WGS) of cell-free DNA (cfDNA) and ichorCNA analysis, we assess tumor fraction (TFx) and genome-wide copy number variation (CNV) patterns, integrated with imaging and biomarker data (18). By describing CNV profiles in this underrepresented patient group, our study provides essential insights into the intrinsic molecular biology of mCRPC and begins to fill the knowledge gap surrounding chemotherapy-naïve patients receiving actinium–lutetium tandem therapy—at a time when PSMA-RPT is rapidly moving earlier in the mCRPC treatment paradigm.

## 2 Materials and methods

### 2.1 Patients

[^225^Ac]Ac-/[^177^Lu]Lu-PSMA-617 was administered as an alternative treatment in accordance with paragraph 37 (‘Unproven Interventions in Clinical Practice’) of the revised Declaration of Helsinki and German medical guidelines (10, 19). Patients provided written informed consent, and the study was approved by the Ethics Committee of University Hospital Heidelberg (S-882/2020). These patients were chemotherapy-naïve, with pretreatment history and diagnostic characteristics summarized in **Tables 1** and **2**. All five patients had received at least androgen deprivation therapy (ADT) prior to [^225^Ac]Ac-/[^177^Lu]Lu-PSMA-617. Patient 1 had received ADT combined with enzalutamide, along with external beam radiation, and presented with osseous and lymphatic metastases. Patient 2 had been treated with ADT followed by radical prostatectomy and lymphadenectomy, and showed bone, hepatic, and lymph node metastases without prior radiation or radioligand therapy. Patient 3 received ADT and enzalutamide, presented with bone metastases, and underwent seeds implantation as well as prior treatment with RaCl_2_. Patient 4 had the most extensive pretreatment history, including ADT in combination with abiraterone and apalutamide, radical prostatectomy with lymphadenectomy, bone and nodal metastases with hepatic involvement, and external radiation, in addition to prior [^177^Lu]Lu-PSMA-617.

**Table 1:** Pre-treatments status of the chemotherapy-naïve patients.

Patient #	Hormonal therapy	Surgery	Metastasis	Radiation	Pre RPT
Patient 1	ADT, Enzalutamide	NA	OSS, LYM	External radiation	NA
Patient 2	ADT	Radical prostatectomy lymphadectomy	OSS, HEP, LYM	NA	NA
Patient 3	ADT, Enzalutamide	NA	OSS	Seeds implantation	RaCl_3_
Patient 4	ADT, Abiraterone, Apalutamide	Radical prostatectomy lymphadectomy	OSS, HEP, LYM	External radiation	[^177^Lu]Lu-PSMA-617
Patient 5	ADT, Abiraterone, Bicalutamide	NA	OSS, LYM	External radiation	NA

ADT, Androgen Deprivation Therapy; NA, Not Applied; OSS (Osseous), Bones; HEP (Hepatic), Liver; LYM (Lymphatic), Lymph nodes.

**Table 2:** Initial diagnosis status of the chemotherapy-naïve patient.

Patient #	Age	ECOG status	iDiagnosis	iGS	iPSA (ng/mL)	iTumor Status
Patient 1	88	1 - 2	2011	3+4 = 7	NA	cT3b cN0 M0 G3
Patient 2	85	1	2008	9	40	pT3a pN1(4/20) cM0 L1 V1
Patient 3	91	3	1996	NA	NA	NA
Patient 4	60	1	2011	4+3 = 7	6.4	pT3a pN0 cM0
Patient 5	85	1	2016	4+3 = 7	7.4	T1c N0 M0 G3

Gleason score 9 for patient 2; primary and secondary patterns not available.

iDiagnosis, initial diagnosis; iGS, initial Gleason score; a/b in GS, Used to differentiate between Gleason patterns within the same score; iPSA, initial PSA; iTumor status, initial tumor status. pT, Pathological / Clinical Tumor stage – extent of primary tumor based on pathology or imaging. pN / cN / N, Pathological / Clinical lymph node involvement. M / cM, Metastasis – clinical or confirmed distant spread of cancer. R0, Surgical margin status – R0 indicates no residual tumor (negative margins); L, Lymphatic invasion; V, Vascular invasion; G, Tumor Grade; NA, Not Available.

Patient 5 had been treated with ADT, abiraterone, and bicalutamide, and presented with bone and lymph node metastases following external radiation. No prior radiopharmaceutical therapy was reported for this patient.

### 2.2 Sample collection and processing

Venous blood samples (20–30 mL) were collected in EDTA tubes and processed within 60 minutes. cfDNA was extracted from plasma using the QIAamp MinElute ccfDNA-Midi Kit. The extracted cfDNA, eluted in 50 µl, was stored at –80 °C until further analysis. Quantification of the extracted cfDNA was performed using the Qubit 1X dsDNA High Sensitivity (HS) Assay Kit (Thermo Fisher, Karlsruhe, Germany) on a Qubit 4 Fluorometer (Thermo Fisher, Karlsruhe, Germany; Q33238). For DNA fragment analysis, including quantification, sizing, and purity determination, the High Sensitivity D1000 ScreenTape assay (Agilent, Waldbronn, Germany) was employed. The cfDNA profile displayed a predominant peak between 100–200 bp, indicative of high-quality mononucleosomal ctDNA appropriate for downstream applications. Data were analyzed using TapeStation Analysis Software 5.1, with 150 bp set as the target fragment size. Libraries were prepared with the Collibri PS DNA Library Prep Kit for Illumina sequencing. Between 10–20 ng of cfDNA input was used for ultra-low pass whole-genome sequencing (ULP-WGS) on a NextSeq instrument with paired-end 2x100 bp reads.

### 2.3 IchorCNA

ULP-WGS data were analyzed with the ichorCNA algorithm (https://github.com/broadinstitute/ichorCNA) in R (v3.3.1) to infer genome-wide copy number alterations and estimate tumor fraction (TFx). The genome was segmented into non-overlapping bins of 1 megabase (Mb), and aligned sequencing reads were quantified within each bin using the HMMcopy Suite. To avoid artifacts, centromeric regions were excluded based on chromosome gap coordinates from the UCSC Genome Browser for hg38 (GRCh38), along with 1 Mb upstream and downstream flanking regions. Normalization of read counts was carried out using the HMMcopy R package, correcting for GC content and mappability biases. Log2 copy number ratios were then calculated for each bin by comparing them to a reference panel of ULP-WGS data from 27 healthy donors. Since cfDNA represents a mixture of tumor- and non-tumor-derived fragments, copy number calling and TFx estimation were performed using a hidden Markov model (HMM) approach. This method assigns discrete copy number states, including hemizygous deletion (HETD, 1 copy), copy-neutral (NEUT, 2 copies), gain (GAIN, 3 copies), amplification (AMP, 4 copies), and high-level amplification (HLAMP, >= 5 copies). Due to resolution limitations, homozygous deletions, which tend to occur at smaller scales than 1 Mb, were not included in the analysis. This algorithm applies a probabilistic framework that segments the genome while simultaneously detecting large-scale CNAs and calculating the proportion of ctDNA (18).

## 3 Results

This study describes baseline genomic and biomarker profiles of five chemotherapy-naïve mCRPC patients upon with [^225^Ac]Ac-/[^177^Lu]Lu-PSMA-617 therapy. Tumor burden, hematological parameters, and ctDNA-derived TFx were integrated with CNA analyses from ULP-WGS. While most patients received only a single treatment cycle, longitudinal ctDNA and biomarker data were available for patient 5 who underwent additional treatment cycles.

### 3.1 Patient 1

**Figure 1:**
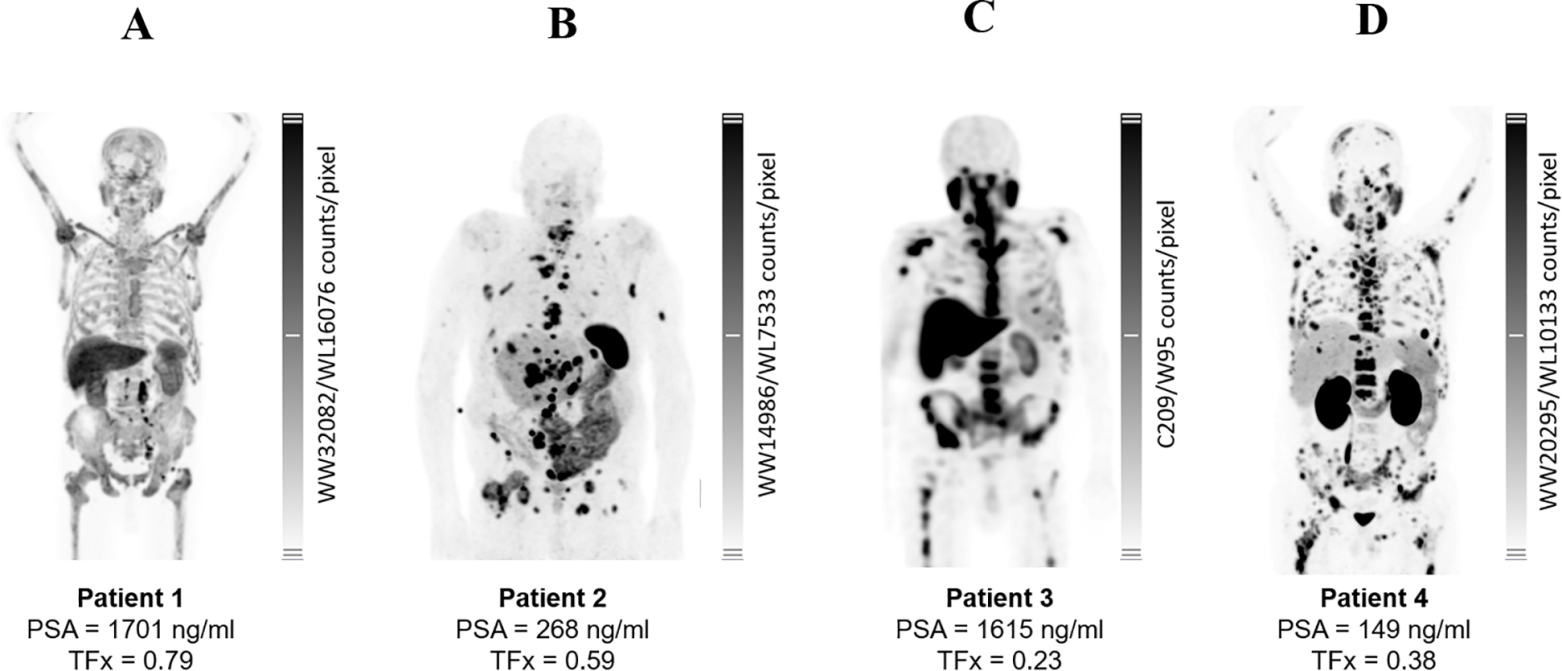
Baseline PSMA-targeted molecular imaging and corresponding serum PSA levels with TFx across four chemotherapy-naïve mCRPC patients. Representative whole-body scans depict heterogeneous metastatic burden and radiotracer distribution at baseline. **(A)** Patient 1 imaged with [^18^F]PSMA-1007 PET demonstrated diffuse osseous and nodal uptake, correlating with markedly elevated serum PSA (1701 ng/ml) and the highest ctDNA tumor fraction (TFx = 0.79). **(B)** Patient 2 underwent [^68^Ga]Ga-PSMA-11 PET, revealing extensive skeletal and visceral disease with PSA of 268 ng/ml and TFx = 0.59. **(C)** Patient 3, staged using [^99m^Tc]Tc-PSMA-GCK01 SPECT, exhibited predominantly skeletal disease a PSA of 1615 ng/mL and comparatively lower TFx = 0.23. **(D)** Patient 4, imaged with [^68^Ga]Ga-PSMA-11 PET, showed widespread but heterogeneous lesions, corresponding to PSA of 149 ng/mL and TFx = 0.38.

Patient 1 presented multiple osseous metastases based on [^18^F]PSMA-1007 PET (**Figure 1A**) and elevated tumor markers, PSA 1701 ng/mL and TFx 0.79, 473 U/L LDH and 426 U/L ALP (**Supplementary Table S1**). Patient 1 was treated with 6 GBq of [^177^Lu]Lu-PSMA-617 and 1 MBq of [^225^Ac]Ac-PSMA-617. CNA profiling revealed whole-chromosome amplifications of 7 and 8, partial amplification of 1p, and small deletions across multiple chromosomes (5, 6, 10, 11, 13, 16, 18, and 20) (**Supplementary Figure S1A**). Renal function appears normal, and leukocyte count is within the expected range. However, hemoglobin is slightly below the reference value (**Supplementary Table S2**).

### 3.2 Patient 2

[^68^Ga]Ga-PSMA-11 PET imaging revealed skeletal, nodal, and visceral metastases (**Figure 1B**). Patient 2 received 6 GBq of [^177^Lu]Lu-PSMA-617 and 2 MBq of [^225^Ac]Ac-PSMA-617. Prior to treatment, Patient 2 exhibited markedly elevated PSA levels (268 ng/mL) and a high TFx (0.59) (**Supplementary Table S3**). Renal function was severely compromised, requiring dialysis and accompanied by anemia. (**Supplementary Table S4**). CNV profiling showed a deletion of chromosome 8p and amplification of chromosome 8q. Additionally, partial amplification of chromosome 9q, partial deletion of chromosome 12p, and amplification of chromosome 12q were observed. Widespread amplifications, were most prominent on chromosomes 1, 5, 7, and 16, while notable losses occurred across chromosomes 2, 3, 4, 6, 13, 15, 16, 17, and 18, (**Supplementary Figure S1B**).

### 3.3 Patient 3

[^99m^Tc]Tc-PSMA-GCK01 SPECT revealed bone marrow carcinomatosis and hepatic involvement (**Figure 1C**). At the initiation of PSMA-RPT, the patient presented with a high tumor burden, reflected by a markedly elevated PSA level of 1615 ng/mL and a TFx of 0.23 (**Supplementary Table S5**). Patient 3 was treated with 4 GBq of [^177^Lu]Lu-PSMA-617 and 4 MBq of [^225^Ac]Ac-PSMA-617. Renal function was moderately impaired and hemoglobin critically low (**Supplementary Table S6**). CNAs included broad gains on the whole chromosome chromosomes 2, 7, 8, 9, 10, 12, and 19, and losses on chromosomes 4, 6, and 16 (**Supplementary Figure S1C**).

### 3.4 Patient 4

Patient 4 demonstrated skeletal, nodal, and visceral metastases on [^68^Ga]Ga-PSMA-11 PET (**Figure 1D**). Biomarkers confirmed active disease (PSA = 149 ng/mL; TFx = 0.38; LDH = 216 U/L; ALP = 418 U/L) (**Supplementary Table S7**). Baseline renal function and hematologic parameters were within the normal range along with stable hematologic functions (**Supplementary Table S8**). Patient 4 received 2 GBq of [^177^Lu]Lu-PSMA-617 and 6 MBq of [^225^Ac]Ac-PSMA-617. CNA profiling revealed a highly unstable genome with widespread amplifications (chromosomes 1–3, 5–8, 10–12, 15, 21, and Y) and deletions across multiple chromosomes (chromosomes 2-6, 8-10, 12, 13, 16, 18, 22) (**Supplementary Figure S1D**).

### 3.5 Patient 5

**Figure 2:**
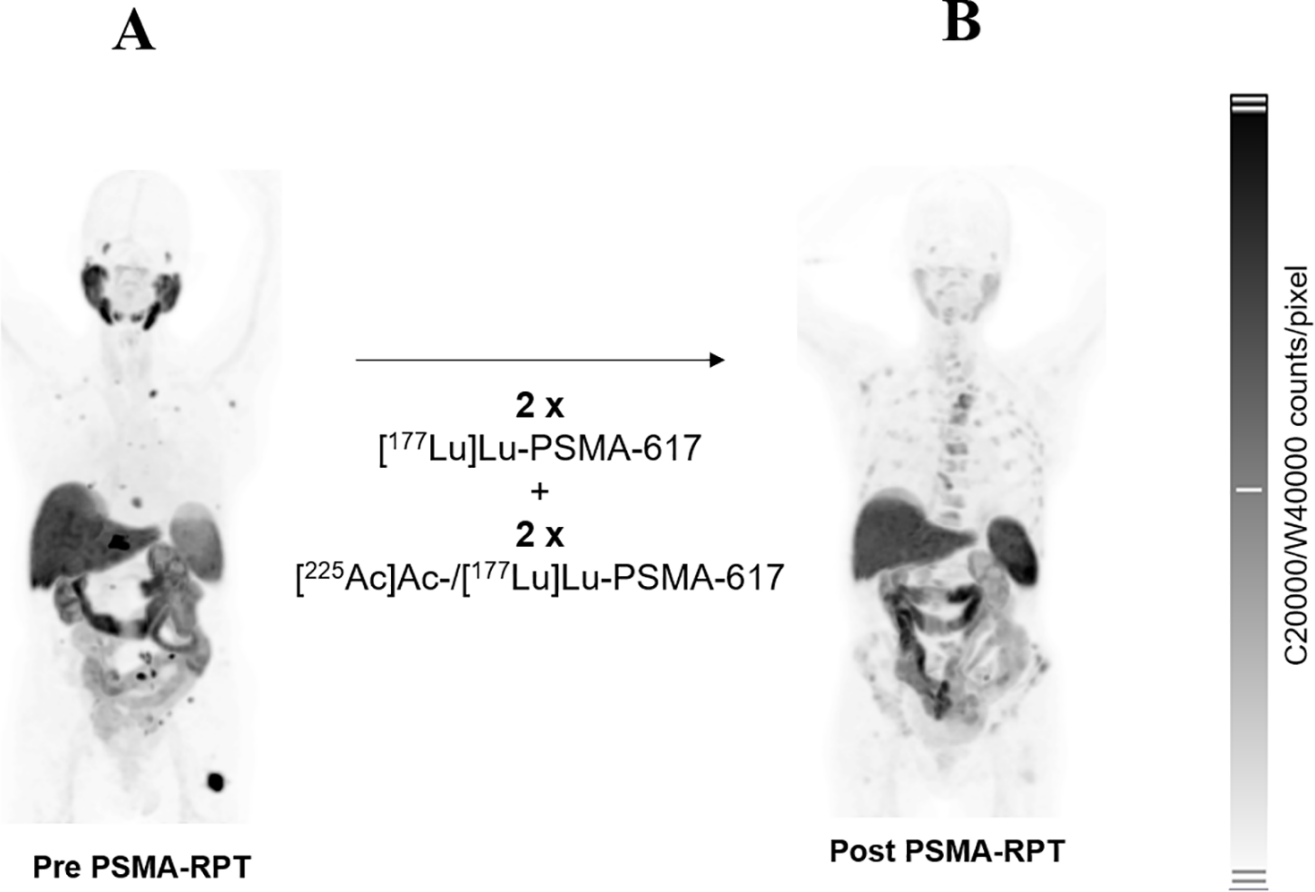
**(A)** Pre- and **(B)** post-treatment [^18^F]PSMA-1007 PET images of Patient 5. The patient received two cycles of 8.5 GBq [^177^Lu]Lu-PSMA-617, followed by two cycles of tandem therapy with 6 GBq [^177^Lu]Lu-PSMA-617 plus 4 MBq [^225^Ac]Ac-PSMA-617, and an additional cycle with 4 GBq [^177^Lu]Lu-PSMA-617 combined with 4 MBq [^225^Ac]Ac-PSMA-617.

**Figure 3:**
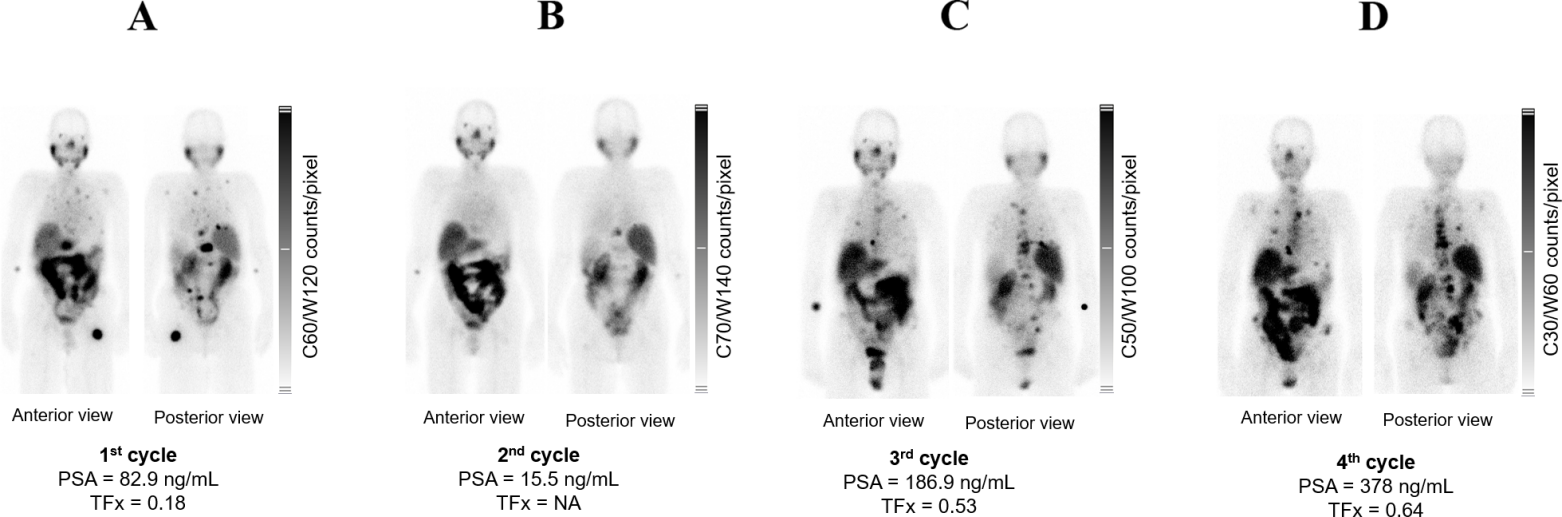
Mid therapy PSMA-RPT images of Patient 5. **(A, B)** display post injection of first two cycles of [^177^Lu]Lu-PSMA-617 monotherapy. Panels **(C, D)** show scans post injection of two cycles of tandem therapy [^225^Ac]Ac-PSMA-617 and [^177^Lu]Lu-PSMA-617. Anterior and posterior views are presented for each panel. PSA and TFx values are indicated for each imaging timepoint.

**Figure 4:**
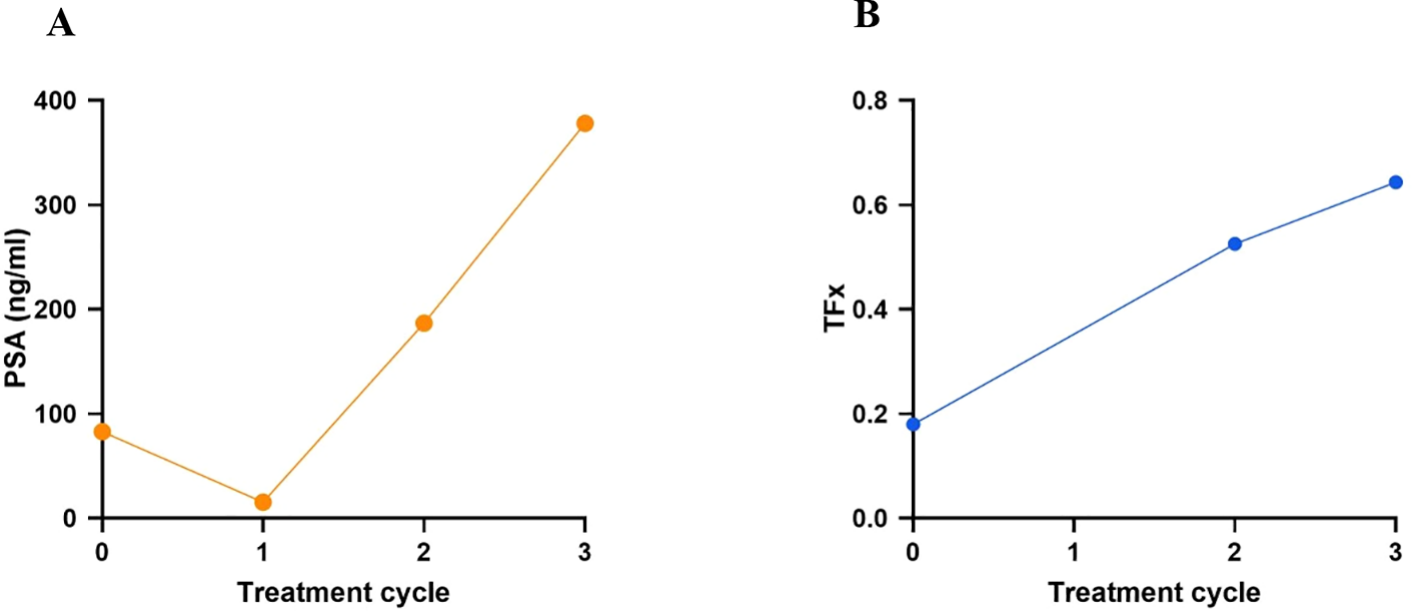
Representative trajectories of PSA **(A)** and TFx **(B)** across three treatment cycles in Patient 5. PSA declined from 82.9 ng/mL at baseline to 15.5 ng/mL after cycle 1, but subsequently rose to 187 ng/mL at cycle 2 and 378 ng/mL at cycle 3. Baseline TFx was 0.18; measurement at cycle 1 was unavailable due to poor venous access. TFx increased from 0.53 at cycle 2 to 0.64 at cycle.

**Figure 5:**
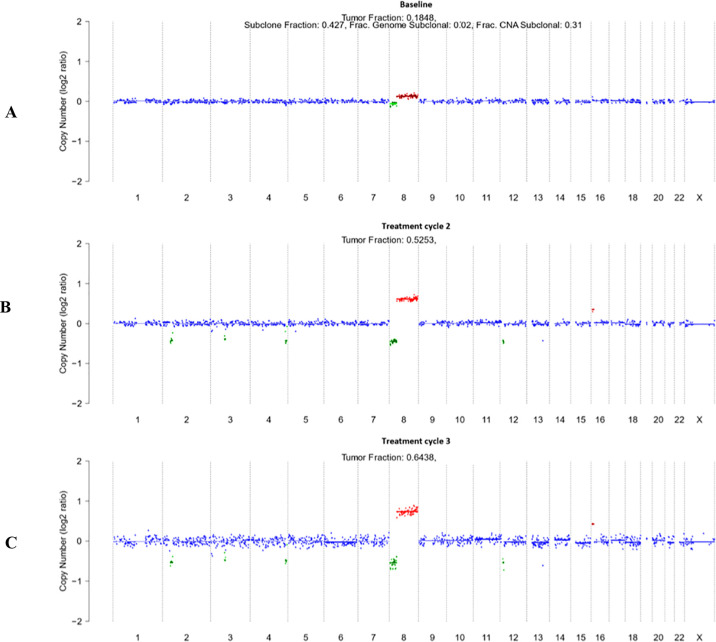
Longitudinal genome-wide CNA profiles from cfDNA in Patient 5 treatment CNV profiles are depicted as log_2_ copy number ratios plotted against genomic coordinates. **(A)** Baseline profile prior to initiation of therapy, showing focal gain on chromosome 8q and loss on 8p. **(B)** Treatment cycle 2 profile, demonstrating persistence of chromosome 8q gain/8p loss, with emergence of additional focal losses on chromosomes 3, 4, and 12. **(C)** Treatment cycle 3 profile, revealing further intensification of chromosome 8q amplification and expansion of CNA events across chromosomes 2, 3, 4, and 12. Data for treatment cycle 1 were not available due to poor venous access. Red indicates copy number gains, green indicates losses, and blue represents neutral copy number segments.

Patient 5 underwent baseline and post-treatment sampling after cycles 2 and 3; cycle 1 sampling was not feasible due to poor venous access. Treatment consisted of two cycles of [^177^Lu]Lu-PSMA-617 (8.5 GBq each) followed by two tandem cycles of [^177^Lu]Lu-PSMA-617 and [^225^Ac]Ac-PSMA-617 (6/4 GBq/MBq and 4/4 GBq/MBq, respectively) (**Figures 2**, **3**). Baseline [^18^F]PSMA-1007 PET demonstrated limited bone involvement (**Figure 2A**), with elevated PSA (82.9 ng/mL), TFx of 0.18, and impaired renal function, while LDH, ALP (**Table 3**; **Figures 4A, B**), hemoglobin, and leukocytes were within normal limits (**Table 4**). Mid-therapy imaging after cycle 1 showed marked response, with PSA decreasing to 15.5 ng/mL (**Table 3**; **Figure 4A**). However, resistance emerged in subsequent cycles: after cycle 2, PSA rose to 186.9 ng/mL, ALP to 104 U/L, and TFx to 0.53, with imaging indicating progression (**Figure 3C**). By cycle 3, PSA further increased to 378 ng/mL and TFx to 0.64, accompanied by worsening renal function (GFR 48.9 mL/min/1.73m², creatinine 1.35 mg/dL) and hematologic decline (**Table 4**). Post-treatment PET confirmed persistent tracer uptake, consistent with non-response (**Figures 2B**, **3D**). CNA profiling revealed focal chromosome 8 amplification at baseline (**Figure 5A**). By cycle 3, these amplifications had intensified and additional deletions appeared on chromosomes 2, 3, 4, and 12 (**Figures 5C, D**).

**Table 3:** Comprehensive overview over the dynamic changes in PSA, TFx, LDH and ALP in Patient 5, following [^225^Ac]Ac-/[^177^Lu]Lu-PSMA-617 regimen.

Tumor markers	Baseline	1^st^ cycle	2^nd^ cycle	3^rd^ cycle	Reference
PSA (ng/mL)	82.9	15.5	187	378	<4
TFx	0.18	NA	0.53	0.64	<0.10
LDH (U/L)	269	236	238	245	<342
ALP (U/L)	85	95	104	155	40–130

**Table 4:** Overview of GFR-CKD-EPI, creatinine, hemoglobin and leukocyte count during [^225^Ac]Ac-/[^177^Lu]Lu-PSMA-617 of Patient 5.

Time point	GFR-CKD-EPI (mL/min/1.73qm)	Creatinine (mg/dL)	Hemoglobin (g/dL)	Leukocyte (G/nL)
Baseline	42.4	1.5	12.8	9.3
1^st^ treatment cycle	40.7	1.6	12.9	11.5
2^nd^ treatment cycle	55.3	1.2	12.0	7.1
3^rd^ treatment cycle	48.9	1.3	11.4	5.0
Reference	≥ 90	0.6–1.4	13–17	4–10

GFR-CKD-EPI, Glomerular Filtration Rate estimated using the Chronic Kidney Disease Epidemiology Collaboration (CKD-EPI) equation, expressed in mL/min/1.73 m².

## 4 Discussion

This exploratory study describes baseline cfDNA-derived CNV profiles from five chemotherapy-naïve patients with mCRPC scheduled to receive [^225^Ac]Ac-/[^177^Lu]Lu-PSMA-617 tandem therapy. Blood samples were collected prior to treatment in four of the five patients, with longitudinal follow-up available only for one individual, Patient 5, who underwent multiple therapy cycles. Although the small sample size and uneven sampling design limit statistical interpretation, this pilot dataset provides an initial descriptive overview of ctDNA-based CNV patterns in a clinically rare patient subset—chemotherapy-naïve individuals scheduled for PSMA-RPT. ctDNA detection though ULP-WGS has been demonstrated as a prognostic tool for monitoring therapy response and assessing the biological behavior of mCRPC (20–22). Patients 1 and 2, both naïve to RPT and chemotherapy, displayed relatively stable CNV profiles with recurrent amplifications in chromosomes previously described in the literature and implicated in prostate cancer (23–25). For example, Patient 1 exhibited amplification of chromosomes 1q and 7, alterations that have been reported in hereditary forms of prostate cancer and associated with more aggressive disease phenotypes (26–30). Furthermore, amplification of chromosome 8, particularly the 8q arm, was a recurrent feature in chemotherapy-naïve patients (Patients 1, 2, and 5). These gains are of particular interest given their frequent association with MYC, a well-characterized oncogene that promotes cell cycle progression, immune evasion, and therapy resistance in mCRPC (25, 31). Recent circulating tumor DNA analyses in PSMA-RPT cohorts have similarly identified 8q amplifications as recurrent features associated with treatment resistance (21). A particularly illustrative case was Patient 5, who underwent serial cfDNA profiling across three timepoints. Remarkably, this patient’s CNV landscape remained stable over time, showing only a persistent loss of 8p and gain of 8q, despite clear clinical progression under RPT. Notably, this patient demonstrated disease progression despite RPT, showing resistance to both ^177^Lu and ^225^Ac, indicating cross-radionuclide resistance. These alterations—frequently reported in large-scale tissue studies of PCa—are known to increase in prevalence during disease progression, particularly in metastatic and hormone-refractory states (24, 32). Although their prognostic impact is not independent of conventional clinical parameters, the consistent presence of 8p loss and 8q gain in this patient suggests that such CNVs may represent early, clonally dominant driver events. Importantly, their stability across serial liquid biopsies strengthens the hypothesis that cfDNA CNV profiling could differentiate fixed, biologically intrinsic genomic alterations from dynamic, therapy-induced genomic changes. Along with chromosome 8 CNA, amplification of chromosome 12q detected in Patient 2, could suggest the involvement of PTPN11 locus (33–35). In the context of our findings, PTPN11 amplification may represent a context-dependent contributor to disease progression in mCRPC, warranting further functional investigation. Moreover, Patient 2 exhibited partial amplification of chromosome 9q, encompassing Tenascin-C (TNC), an extracellular matrix glycoprotein that facilitates tumor invasion and metastasis by promoting cell migration and tumor–stroma interaction (36, 37). In contrast, Patients 3 and 4, while also chemotherapy-naïve, had received prior RPT (RaCl_3_ and/or [^177^Lu]Lu-PSMA-617). Their ctDNA profiles revealed markedly more disrupted CNV landscapes, with irregular patterns and structural complexity absent in RPT-naïve patients, suggesting that prior exposure to ionizing radiation—even without chemotherapy—may leave a lasting ‘genomic scar’ through DNA repair processes such as non-homologous end joining (NHEJ) (38–40). Taken together, although constrained by the very small sample size and lack of serial monitoring in most cases, these observations offer a preliminary window into the genomic architecture of chemotherapy-naïve mCRPC patients at the onset of PSMA-RPT. Given the complex and heterogeneous nature of mCRPC, integrating multi-modal liquid biopsy approaches—including serial cfDNA CNV profiling and CTC/EV-based PSMA protein detection—may provide a more comprehensive assessment of disease biology and improve patient stratification for PSMA-targeted therapies (41). Recurrent CNV alterations—including amplifications in chromosomes 1, 7, 8, 9, and 12—emerge as descriptive features worth further investigation. However, their biological significance, temporal dynamics, and potential links to treatment response or resistance remain to be clarified through expanded, longitudinal studies.

## 5 Conclusion

While limited by a small cohort, this exploratory study provides valuable initial insights into the genomic landscape of chemotherapy-naïve mCRPC patients undergoing tandem [^225^Ac]Ac-/[^177^Lu]Lu-PSMA-617 therapy. The identification of recurrent, stable copy number alterations—particularly involving chromosomes 8p and 8q—suggests potential intrinsic biomarkers of disease biology and treatment resistance. These findings underscore the promise of cfDNA-based genomic profiling as a non-invasive tool to guide therapeutic stratification and optimize PSMA-RPT outcomes. Future larger and longitudinal studies are warranted to validate these genomic signatures and fully elucidate their clinical utility for improving personalized treatment strategies in advanced prostate cancer.

## Data Availability

All data analyzed in this case study are presented within the article. Anonymized clinical datasets from patients are available from the corresponding author upon reasonable request.
