# Supplementary material for: Genomic analysis in chemotherapy-naïve prostate cancer prior to PSMA-targeted treatment
Source: Front Oncol. 2026 Feb 4;16:1741080. doi: 10.3389/fonc.2026.1741080 (PMC12913125; doi:10.3389/fonc.2026.1741080)
Supplement: Supplementary file 1 [file DataSheet1.docx]

Supplementary Material

# Supplementary Data

**Supplementary Table 1.** Comprehensive overview of PSA, TFx, LDH and ALP in Patient 1, following [^225^Ac]Ac-/[^177^Lu]Lu-PSMA-617 regimen.

| Tumor Markers | Baseline | Reference |
| --- | --- | --- |
| PSA (ng/mL) | 1701 | <4 |
| TFx | 0.79 | >0.10 |
| LDH (U/L) | 473 | <342 |
| ALP (U/L) | 426 | 40–130 |

**Supplementary Table 2.** Baseline assessment of GFR-CKD-EPI, creatinine, hemoglobin and leukocyte count during [^225^Ac]Ac-/[^177^Lu]Lu-PSMA-617 of Patient 1.

| Time point | GFR-CKD-EPI (mL/min/1.73qm) | Creatinine (mg/dL) | Hemoglobin (g/dL) | Leukocyte  (G/nL) |
| --- | --- | --- | --- | --- |
| Baseline | 89.4 | 0.66 | 9.7 | 4.8 |
| Reference | ≥ 90 | 0.6–1.4 | 13–17 | 4–10 |

GFR-CKD-EPI: Glomerular Filtration Rate estimated using the Chronic Kidney Disease Epidemiology Collaboration (CKD-EPI) equation, expressed in mL/min/1.73 m².

**Supplementary Table 3.** Comprehensive overview of PSA, TFx, LDH and ALP in Patient 2, following [^225^Ac]Ac-/[^177^Lu]Lu-PSMA-617 regimen.

| Tumor Markers | Baseline | Reference |
| --- | --- | --- |
| PSA (ng/mL) | 268 | <4 |
| TFx | 0.59 | <0.10 |
| LDH (U/L) | NA | <342 |
| ALP (U/L) | NA | 40–130 |

**Supplementary Table 4.** Baseline assessment of GFR-CKD-EPI, creatinine, hemoglobin and leukocyte count during [^225^Ac]Ac-/[^177^Lu]Lu-PSMA-617 of Patient 2.

| Time point | GFR-CKD-EPI (mL/min/1.73qm) | Creatinine (mg/dL) | Hemoglobin (g/dL) | Leukocyte  (G/nL) |
| --- | --- | --- | --- | --- |
| Baseline | 18.6 | 2.9 | 8.7 | 8.5 |
| Reference | ≥ 90 | 0.6–1.4 | 13–17 | 4–10 |

GFR-CKD-EPI: Glomerular Filtration Rate estimated using the Chronic Kidney Disease Epidemiology Collaboration (CKD-EPI) equation, expressed in mL/min/1.73 m².

**Supplementary Table 5.** Comprehensive overview of PSA, TFx, LDH and ALP in Patient 3, following [^225^Ac]Ac-/[^177^Lu]Lu-PSMA-617 regimen.

| Tumor Markers | Baseline | Reference |
| --- | --- | --- |
| PSA (ng/mL) | 1615 | <4 |
| TFx | 0.23 | <0.10 |
| LDH (U/L) | 409 | <342 |
| ALP (U/L) | 1348 | 40–130 |

**Supplementary Table 6.** Baseline assessment of GFR-CKD-EPI, creatinine, hemoglobin and leukocyte count during [^225^Ac]Ac-/[^177^Lu]Lu-PSMA-617 of Patient 3.

| Time point | GFR-CKD-EPI (mL/min/1.73qm) | Creatinine (mg/dL) | Haemoglobin (g/dL) | Leukocyte  (G/nL) |
| --- | --- | --- | --- | --- |
| Baseline | 39.1 | 1.5 | 6 | 6.4 |
| Reference | ≥ 90 | 0.6–1.4 | 13–17 | 4–10 |

*GFR-CKD-EPI: Glomerular Filtration Rate estimated using the Chronic Kidney Disease Epidemiology Collaboration (CKD-EPI) equation, expressed in mL/min/1.73 m².*

**Supplementary Table 7.** Comprehensive overview of PSA, TFx, LDH and ALP in Patient 4, following [^225^Ac]Ac-/[^177^Lu]Lu-PSMA-617 regimen.

| Tumor Markers | Baseline | Reference |
| --- | --- | --- |
| PSA (ng/mL) | 149.4 | <4 |
| TFx | 0.38 | <0.10 |
| LDH (U/L) | 216 | <342 |
| ALP (U/L) | 418 | 40–130 |

**Supplementary Table 8**. Overview of GFR-CKD-EPI, creatinine, hemoglobin and leukocyte count during [^225^Ac]Ac-/[^177^Lu]Lu-PSMA-617 of Patient 4.

| Time point | GFR-CKD-EPI (mL/min/1.73qm) | Creatinine (mg/dL) | Hemoglobin (g/dL) | Leukocyte  (G/nL) |
| --- | --- | --- | --- | --- |
| Baseline | 102 | 0.73 | 10.9 | 7.2 |
| Reference | ≥ 90 | 0.6 – 1.4 | 13-17 | 4-10 |

*GFR-CKD-EPI: Glomerular Filtration Rate estimated using the Chronic Kidney Disease Epidemiology Collaboration (CKD-EPI) equation, expressed in mL/min/1.73 m².*


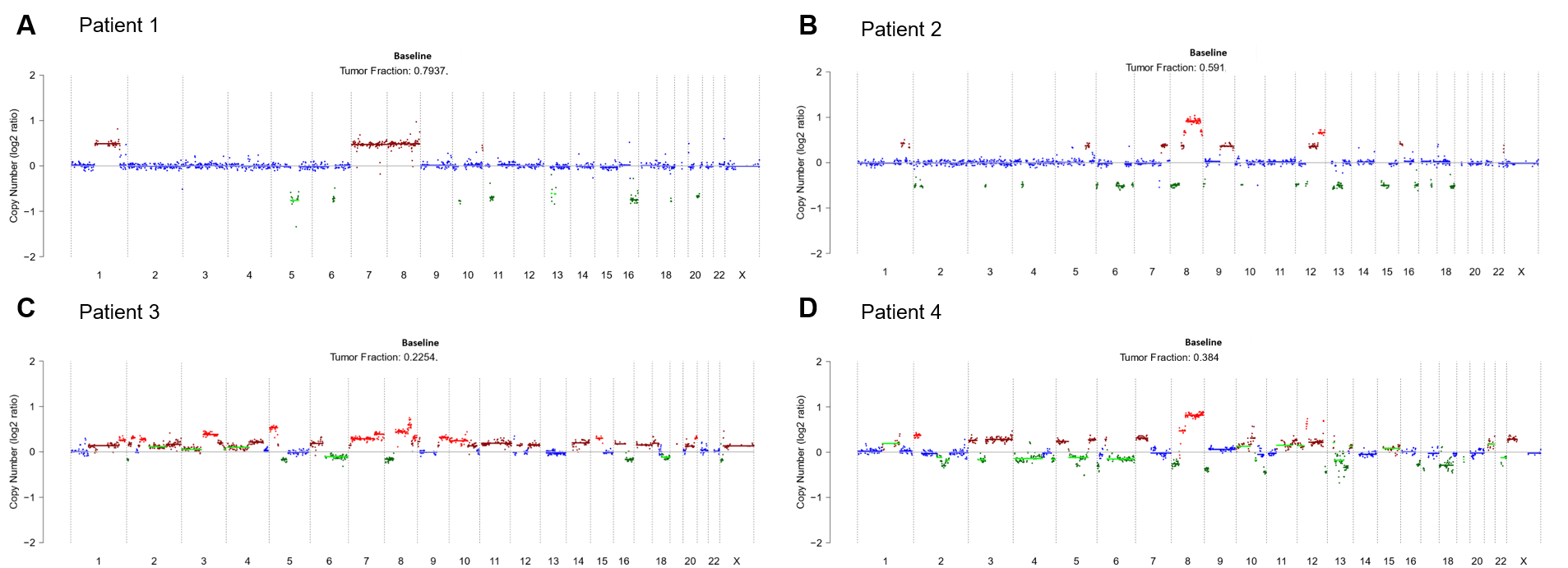


**Supplementary Figure 1.** Genome-wide CNA profiles from baseline cfDNA in four mCRPC patients prior to tandem [^225^Ac]Ac-/[^177^Lu]Lu-PSMA-617 therapy. CNA profiles were generated from ULP-WGS and analyzed with ichorCNA. CNA profiles are depicted as log2 copy number ratios plotted against genomic coordinates Red indicates copy number gain, green indicates copy number loss, and blue represents neutral copy number regions. All profiles are shown at baseline (pre-treatment) to represent the intrinsic or treatment-altered genomic state before initiation of tandem PSMA-RPT.
